# Developing a Novel, At-Home Procedure Curriculum for Fourth-Year Medical Students in Response to the COVID-19 Pandemic

**DOI:** 10.7759/cureus.15215

**Published:** 2021-05-24

**Authors:** Joel Gieswein, Robert Tennill, Richard Austin, Kristin Delfino, Danuta Dynda, Sharon Kim

**Affiliations:** 1 Emergency Medicine, Southern Illinois University School of Medicine, Springfield, USA; 2 Center for Clinical Research, Southern Illinois University School of Medicine, Springfield, USA

**Keywords:** low-fidelity models, low-cost task trainers, curriculum development, simulation in medical education, halo procedures, lateral canthotomy, landmark-guided pericardiocentesis, surgical cricothyrotomy, landmark-guided subclavian central venous catheterization

## Abstract

The coronavirus disease 2019 (COVID-19) pandemic provided our institution a unique opportunity to develop a new procedural curriculum for our fourth-year, emergency medicine-bound medical students. A significant portion of our ED’s fourth-year elective has traditionally been centered in our simulation center, using high-fidelity simulation models to practice important emergency medicine procedures. Due to the pandemic, the simulation center was unavailable for our use, and this new curriculum was created in an effort to fill this gap in procedural education.

## Introduction

Simulation models are utilized in medical education to practice invasive procedures prior to learners performing the procedures on patients. These models range in their ability to accurately simulate the human body, where a highly representative model is termed high-fidelity and a less accurate model as low-fidelity [1]. There is a significant question regarding the incremental, educational value of higher-cost, high-fidelity models in the literature as compared to lower-cost task trainers [2]. In fact, some sources recommend a focus on improving the functional fidelity of a model or task trainer over anatomic fidelity [3].

The coronavirus disease 2019 (COVID-19) pandemic led to many restrictions on gatherings, causing the majority of schools and universities to suspend in-person activities and move their classes online. Due to the pandemic, the Department of Emergency Medicine at our institution also modified our curriculum to allow medical students to continue their education on an online platform.

The majority of emergency physicians (76.3% according to one post-ConCert examination survey) work in community hospital settings, where knowledge and continued practice of rare procedures remain important after residency graduation [4]. Access to a simulation center at community hospitals is not guaranteed, so the current pandemic provides the opportunity to create an applicable curriculum for medical students that they can continue to use after residency. Certain critical emergency procedures are considered “high-acuity, low-opportunity” (HALO) as described by Chiniara et al. [5]. HALO procedures include pericardiocentesis, lateral canthotomy, emergency thoracotomy, and surgical cricothyrotomy.

Others have previously described methods of creating low-fidelity models at home, then utilizing them for personal practice or as a wilderness medicine curriculum [6-7]. We sought to teach fourth-year medical students how to perform HALO procedures by creating a set of procedural models from household components with accompanying educational and technical information to build and use these models. This new curriculum was provided as a substitute for the in-person procedure curriculum traditionally used for the Emergency Medicine (EM) Residency Readiness elective at our institution.

## Materials and methods

**Figure 1:**
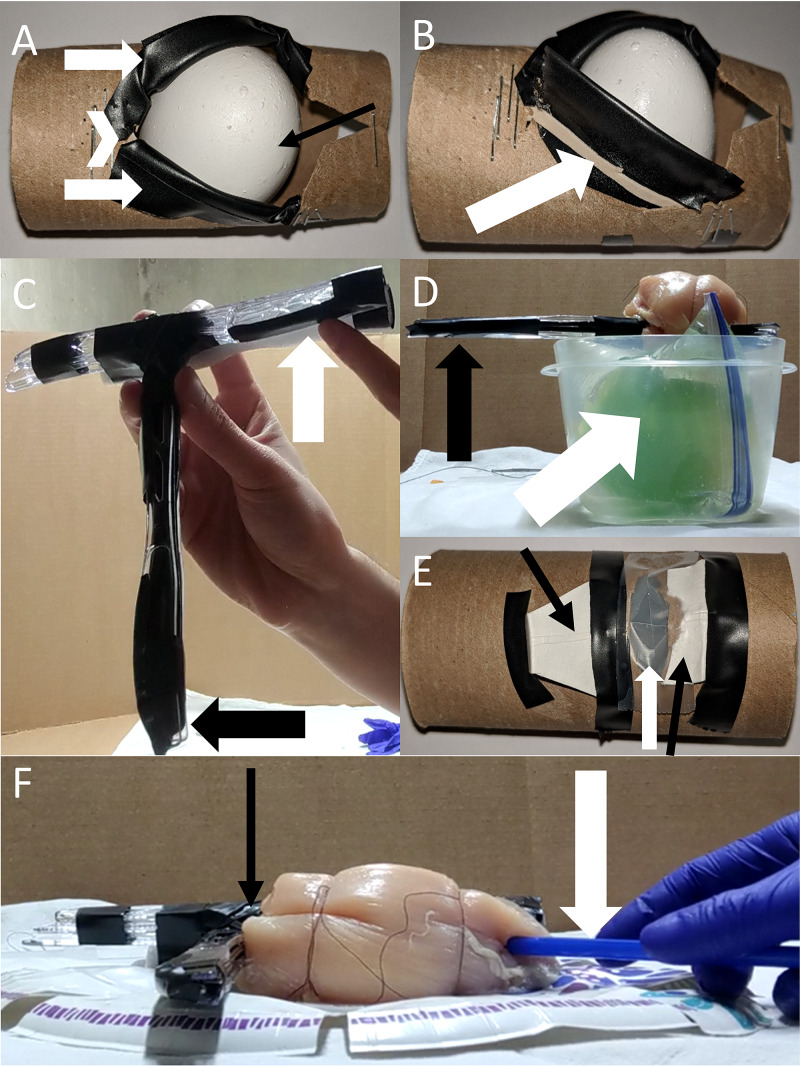
Low-fidelity procedural models created from common household items. (A) Lateral canthotomy model. A toilet paper tube was used to simulate the orbit with electrical tape for eyelids (white arrows) meeting at the lateral canthus (white chevron) and an egg for the eye (black arrow). (B) Oblique view of lateral canthotomy model with tape reflected showing rubber band (white arrow) representing canthal tendons. (C) Plastic knives were secured together to simulate sternum and clavicle/ribs (black arrow) for the pericardiocentesis and subclavian models. Hollowed-out backer rod (white arrow) simulated subclavian vein. (D) Pericardiocentesis model with plastic knife assembly (black arrow) representing ribs and sternum over a plastic zip-top bag (white arrow) which contains a gelatin-filled balloon surrounded by water. (E) Surgical cricothyrotomy model made from the opposite side of toilet paper tube from lateral canthotomy model. Secured to the model are pieces of paper plate to simulate cartilage (black arrows) surrounding a cellophane tape-covered hole (white arrow) which simulates cricothyroid membrane. Napkins were layered over this assembly to simulate the skin and fascial layers. (F) The subclavian model with plastic knife sternum/clavicle assembly covered by a chicken breast (black arrow), with a beverage straw being used as the catheter (white arrow).

We created a curriculum that used low-fidelity models to teach 11, fourth-year medical students enrolled in our EM Residency Readiness elective how to perform three HALO procedures: lateral canthotomy (Figures 1A, 1B), landmark-guided pericardiocentesis (Figures 1C, 1D), and surgical cricothyrotomy (Figure 1E). A fourth procedure, landmark-guided subclavian central venous catheterization (CVC) (Figure 1C, 1F), was included in the curriculum due to the importance of emergent vascular access in the EM physician’s procedural repertoire and the feasibility of creating such a model.

The curriculum was administered to the 11 students in three parts. Pre-recorded videos were created to demonstrate each procedure with instructions on building a functioning low-fidelity model. Written guides explained the procedures and listed possible materials that could be utilized to make the models. Finally, live demonstrations via video conference software demonstrated the procedures in real-time and answered questions from students.

We assessed the effectiveness of our curriculum using before and after course surveys. We asked each student to rate their perceived confidence on a 5-point Likert scale in the performance of the procedures taught and asked process improvement questions regarding video quality and suggestions for modifications. Statistical analysis was performed using Fisher’s exact test on a calculation of percentage change of perceived confidence before and after the curriculum.

## Results

**Figure 2:**
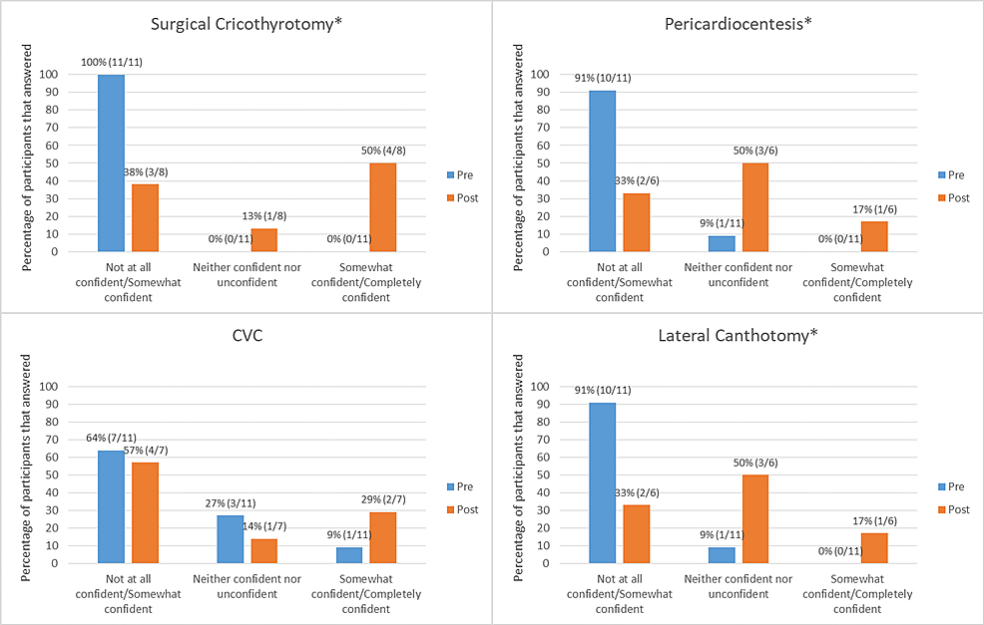
Measure of confidence for each low-fidelity procedure performed. CVC: Subclavian central venous catheterization. *p < 0.05.

We found that there was a statistically significant correlation between improvement in confidence performing the procedure and exposure to the curriculum for the surgical cricothyrotomy (p = 0.0048), pericardiocentesis (p = 0.0048), and lateral canthotomy (p = 0.0276) procedures, but not for the subclavian CVC procedure (p = 0.6391) (Figure 2).

## Discussion

Some limitations to our assessment of the course include a lower response rate to the post-course survey than to the pre-course survey, 11 responses pre-course, and six complete responses post-course (two additional respondents did not complete all sections post-course). Additionally, we were unable to match responses between the two surveys. It is possible that students who did not complete the post-course survey did not find their confidence level increased with their participation in this curriculum.

Possible confounders of our data include prior knowledge or background of participants (which was not measured a priori), a small sample size of students, and only one class level of medical students included from our single medical school. In addition, due to the pandemic restrictions in April of 2020 when the course was held, we were unable to meet with the students in person and thus course instructors could not effectively assess student competency. Future studies could improve on each of these limitations of this current study.

Time was a significant limiting factor in producing a three-part curriculum such as this. However, most aspects of the models were relatively simple to assemble and used materials commonly found in one’s home, such as electrical tape, plastic knives, craft wire, balloons, gelatin, toilet paper tubes, steak knives, sewing needles, paper plates, and rubber bands. Fidelity can be improved with the use of a 14-gauge angiocath and 10 mL syringe when available for the subclavian CVC model. One suggestion was to distribute the curriculum in advance so that students could have models prepared to follow along in real-time with live sessions. Additionally, improving the quality of video recordings was a commonly identified area of improvement from students for the curriculum.

Despite these limitations, we note a significant improvement in self-perceived confidence performing the three HALO procedures after exposure to our curriculum. Future areas of study may include comparison with students participating in a more traditional high-fidelity simulation curriculum or whether a structured curriculum that is self-guided may be more effective. Additionally, this type of curriculum could be extended to other medical specialties and to other levels of learners. This type of curriculum extension would allow for serial evaluations of learner's progress in addition to enlarging sample size.

## Conclusions

Our curriculum can easily be scaled, as the only limits are the materials the learner has available to create the models and time. It could be used as a supplement to a formal high-fidelity procedure curriculum or expanded with other procedures. Additionally, this curriculum could be utilized by practicing emergency physicians to maintain their skills or by any level of learner seeking increased procedural proficiency. In the post-COVID-19 era of medical education, curricula utilizing low-fidelity task trainers are likely to become an important component to achieve the highest procedural competence for our learners.
